# Identification of Ferroptosis-Related Genes Associated With Cryptorchidism via Bioinformatics and Experimental Verification

**DOI:** 10.1155/genr/7355474

**Published:** 2025-05-23

**Authors:** Tian Du, Yifeng Ge, Zheng Zhou, Jun Jing, Yuming Feng, Hualong Ding, Jinzhao Ma, Bing Yao

**Affiliations:** ^1^Center of Reproductive Medicine, Jinling Hospital, Affiliated Hospital of Medical School, Southeast University, Nanjing, Jiangsu, China; ^2^Center of Reproductive Medicine, Jinling Hospital, Affiliated Hospital of Medical School, Nanjing University, Nanjing, Jiangsu, China

**Keywords:** BRDT, cryptorchidism, ferroptosis, GEO, PARP11, WGCNA

## Abstract

**Objectives:** Cryptorchidism is a notorious innate malformation in children that always leads to oligospermatism or azoospermatism. Moreover, there is a relationship between oxidative stress and spermatogenesis dysfunction caused by cryptorchidism. Ferroptosis is associated with iron metabolism and oxidative stress as a novel form of cell death regulation, which is involved in the pathogenesis of many diseases. Hence, ferroptosis may play an important role in spermatogenesis dysfunction in case of cryptorchidism. Therefore, the purpose of this study was to identify the key ferroptosis-related genes that influence spermatogenesis in patients with cryptorchidism and provided new strategies for the prevention and treatment of spermatogenesis dysfunction in cryptorchidism patients in clinical practice.

**Methods:** Gene expression information was downloaded from the Gene Expression Omnibus (GEO) and ArrayExpress databases. The differentially expressed genes (DEGs) were selected using the limma R package. Next, one crucial module, Maroon, was identified via Weighted Gene Coexpression Network Analysis (WGCNA). Ferroptosis-related genes were downloaded from FerrDb v2 database. GO and KEGG analyses were subsequently conducted. Moreover, these differentially expressed ferroptosis-related genes (DE-FRGs) were intersected with the DEGs of AdPlus/AdMinus. Two key genes most closely associated with spermatogenesis dysfunction in cases of cryptorchidism were subsequently identified. Furthermore, immunohistochemistry (IHC) and Receiver Operating Characteristic (ROC) analyses were conducted to validate our conclusions. Finally, miRWalk3.0 and TargetScan were used to predict the pivotal target microRNAs.

**Results:** One critical module and two hub genes that are strongly related to the pathogenesis of spermatogenesis dysfunction in patients with cryptorchidism were identified. Gene Set Enrichment Analysis, ROC and IHC analyses were conducted and the results revealed that BRDT and PARP11 might play critical roles in spermatogenesis dysfunction in patients with cryptorchidism.

**Conclusion:** Our study identified two ferroptosis-related genes, BRDT and PARP11 might play a role in the pathogenesis of spermatogenesis dysfunction in patients with cryptorchidism, which provided a novel perspective for the prevention and treatment of spermatogenesis dysfunction in patients with cryptorchidism in clinical practice.

## 1. Introduction

Cryptorchidism is a well-known congenital malformation in children, that usually leads to clinical infertility in males due to oligospermatism or azoospermatism [1, 2]. Cryptorchidism is defined as unilateral testis that neither resides nor can be manipulated into the scrotum [3]. The pathological changes caused by cryptorchidism include the inhibition of spermatogonial differentiation, spermatogenesis retardation, seminiferous tubule atrophy, germ cell depletion and fibrosis, and a decrease in the relative weight of the testis [4, 5]. The germ cells in the male testicles need to be located in the scrotum at a temperature slightly lower than the body temperature to develop normally, whereas the testicles of patients with cryptorchidism are trapped in the inguinal canal or in the abdominal cavity. Therefore, the testicles are in a relatively high temperature environment, inducing an increase in intracellular ROS levels, which results in the obstruction of spermatogenesis [6, 7]. Spermatocytes and spermatids are the most sensitive cell types affected by heat stress [8, 9]. Previous studies have shown that spermatogenesis dysfunction in cryptorchid patients are closely related to apoptosis [10, 11]. Moreover, there has been an increasing trend in the morbidity of congenital cryptorchidism based on recent studies [12–14]. Thus, it is critical to investigate the molecular mechanisms of spermatogenesis dysfunction in patients with cryptorchidism in and improve the treatment efficacy and prognosis of these patients.

Ferroptosis is a newly identified form of regulated cell death form that dysregulates cellular tissue homeostasis, and characterized by the iron-dependent accumulation of lipid peroxides to lethal levels [15]. Additionally, it is a newly defined mode of cell death, that is extremely different from other modes, such as necroptosis or autophagy. Moreover, ferroptosis is connected with various male reproductive diseases, including testicular dysgenesis, spermatogenesis dysfunction, and blood-testis barrier (BTB) disruption [16–19]. However, whether ferroptosis is involved in spermatogenesis dysfunction in individuals with cryptorchidism is unknown.

In this study, we identified differentially expressed genes (DEGs) between cryptorchid and control samples via data mining analytic techniques. These DEGs were subsequently intersected with the ferroptosis dataset to access differentially expressed ferroptosis-related genes (DE-FRGs). Furthermore, key DE-FRGs that may interfere with spermatogenesis in individuals with cryptorchidism were investigated to identify vital genes.And we delved into the pathogenesis of spermatogenesis dysfunction of individuals with cryptorchidism at the molecular level. In summary, our results contribute to the understanding of the possible role of ferroptosis in spermatogenesis dysfunction and provide new perspectives on prevention and treatment of the infertility in patients with cryptorchidism.

## 2. Materials and Methods

### 2.1. Microarray Data Extraction and Data Processing

**Figure 1:**
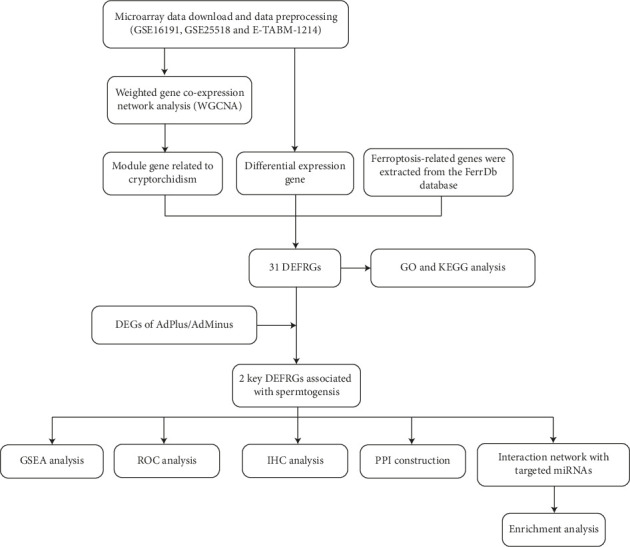
The workflow chart of data preparation, processing, analysis, and validation.

Gene expression information on cryptorchidism and spermatogenesis was downloaded from the Gene Expression Omnibus (GEO) (https://www.ncbi.nlm.nih.gov/geo/) and ArrayExpress (https://www.ebi.ac.uk/biostudies/arrayexpress/) databases, including GSE16191, GSE25518 and E_TABM_1214, all of which use Affymetrix Chip and are available on the GPL570 platform. The data processing was executed as previously described [20]. In brief, the “inSilicoMerging” R package was used for matrix-merging and the “sva” R package was used to correct for batch effects to ensure that the median signal expression intensity of each sample was roughly at the same level. The flowchart for this research process is shown in Figure 1.

### 2.2. Identification of DEGs

DEGs identification were carried out via the “limma” R package. To acquire more DEGs, |FC| > 1.5 and *p* value < 0.05 were set as the thresholds. DEGs were visualized using R software, includeing heatmap and volcano plots.

### 2.3. Weighted Gene Co-Expression Network Analysis (WGCNA)

The WGCNA was proceeding as previously described [21, 22]. Briefly, all genes expressed in the cryptorchid and control samples were sequenced in accordance with median absolute deviation (MAD) values, and the top 50 percent of the largest genes of MAD were chosen for WGCNA. First, for all pair-wise genes, Pearson's correlation matrices and the average linkage method were employed. A weighted adjacency matrix was subsequently created, and we chose the power of 12 as the soft-thresholding parameter. Soon afterward, a weighted adjacency matrix was built by the power function. We subsequently changed the adjacency matrix into a topological overlap matrix (TOM), after which the corresponding dissimilarity (1-TOM) was determined. The sensitivity was set to 3. Next, hierarchical clustering was executed to confirm the modules, and the eigengenes were calculated. Finally, to identify cryptorchidism-related modules, we estimated the relevance between each module and phenotype via Pearson's correlation analysis.

### 2.4. Extraction of Ferroptosis-Related Genes From FerrDb

Ferroptosis-related genes were downloaded from the FerrDb v2 database (http://www.zhounan.org/ferrdb/current/).

### 2.5. GO and KEGG Analyses

Three aspects of GO functional enrichment analysis, biological process (BP), cellular component (CC) and molecular function (MF) were performed. The KEGG analysis was also carried out. To determine the biological functions of the genes and associated pathways, the “clusterProfiler” R package was used to conduct enrichment analysis [23]. Items with *p* values less than 0.05 were found to be significant.

### 2.6. Construction and Evaluation of the Receiver Operating Characteristic (ROC) Curve

ROC analysis curves were delineated by the “pROC” R package to indicate the diagnostic efficacy of the key DE-FRGs [24].

### 2.7. Gene Set Enrichment Analysis (GSEA)

GSEA was used to assess the distribution of genes in the high expression group (≥ 50%) or low expression group (< 50%) to determine their contribution to phenotype or functional signaling pathways, with the gene set ranging 5 to 5000, and 1000 resampling [25].

### 2.8. Protein-Protein Interaction (PPI) Construction and Gene–miRNA Interaction Networks

GeneMANIA was used to perform PPI analysis of the two hub genes and their 20 interacting genes [26]. miRWalk (http://mirwalk.umm.uni-heidelberg.de/) and TargetScan (https://www.targetscan.org) were used to predict pivotal target miRNAs, and the key gene–miRNA interaction networks were produced via Cytoscape (13).

### 2.9. Animals and Tissue Preparation

Immature male Sprague-Dawley rats (22 days of age) were purchased from Jinan Pengyue Experimental Animal Breeding Co., Ltd. The rats were anesthetized to generate one-sided cryptorchidism and a small wound was made in the midsection of each individual. The testis was displaced into the abdomen and secured to the abdominal wall. Finally, we closed the wound carefully and disinfected the surrounding area. Then, the rats were housed with free access to food and water under a 12-h light: 12-h dark and 22 ± 2°C environment. The animals were killed by cervical dislocation at 30 days after surgery and the testes were removed. The animal experiments were approved by the Ethics Committee of Nanjing Jinling Hospital (approval number: 2020JLHGKJDWLS-173 of December 15^th^, 2020).

### 2.10. Testicular Histopathological and Immunohistochemistry (IHC) Analysis

The testes were fixed in 4% paraformaldehyde for 24 h, embedded in paraffin, and cut into 5 μm thick paraffin sections. Then, the slides were stained with hematoxylin solution and eosin solution. Rabbit anti-BRDT antibodies (1:500, Cat. ab288435, Abcam) and rabbit anti-poly(ADP-ribose) polymerase (PARP)11 (1:100, Cat. bs-19885R, Bioss) were used as primary antibodies, and antirabbit IgG was used as a secondary antibody (1:2,000, Cat. ab205718, Abcam). Images were obtained via optical microscopy.

### 2.11. Statistical Analysis

R and GraphPad software programs were used to analyze these data. Student's *t*-test was used for DEGs analysis to compare the differences between the cryptorchid and control samples. Differences were considered statistically significant when the *p* value was less than 0.05.

## 3. Results

### 3.1. DEGs Between Cryptorchidism and Control Samples

**Figure 2:**
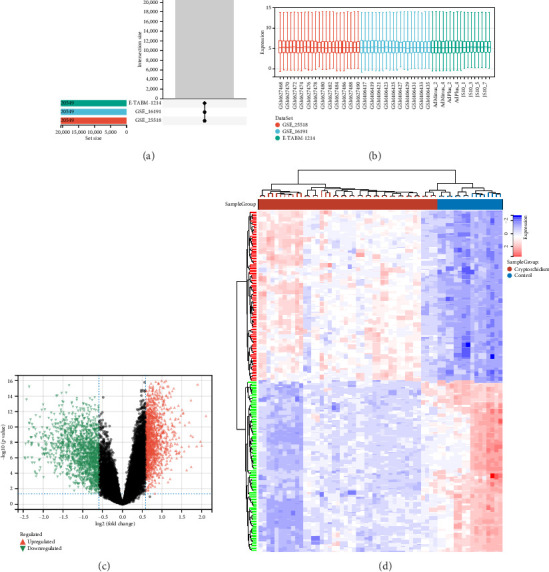
Identification of the ferroptosis DEGs in cryptorchidism. (a) The same gene probes were used in three datasets. (b) Normalization of gene expression data in samples. (c) Volcano plot. Red dots represent upregulated genes, black dots represent nonsignificant genes, and green dots represent downregulated genes (|FC| ≥ 1.5 and *p* values < 0.05). (d) The heat map of the cryptorchidism-related gene expression between cryptorchidism samples and control samples.

The gene expression data were downloaded from the GSE16191, GSE25518 and E_TABM_1214 datasets in the GEO and ArrayExpress databases. The three datasets were combined and normalized to eliminate the batch differences and abnormal samples (Figures 2(a), 2(b)). Differential gene expression analysis revealed a total of 2872 DEGs, including 1334 upregulated genes and 1538 downregulated genes (|FC| ≥ 1.5 and *p* values < 0.05) (Figure 2(c)). The *p* values of the DEGs were corrected and a heatmap was generated to visualize the expression of the DEGs between the cryptorchidism and control samples (Figure 2(d)).

### 3.2. WGCNA and Identification of Critical Modules

**Figure 3:**
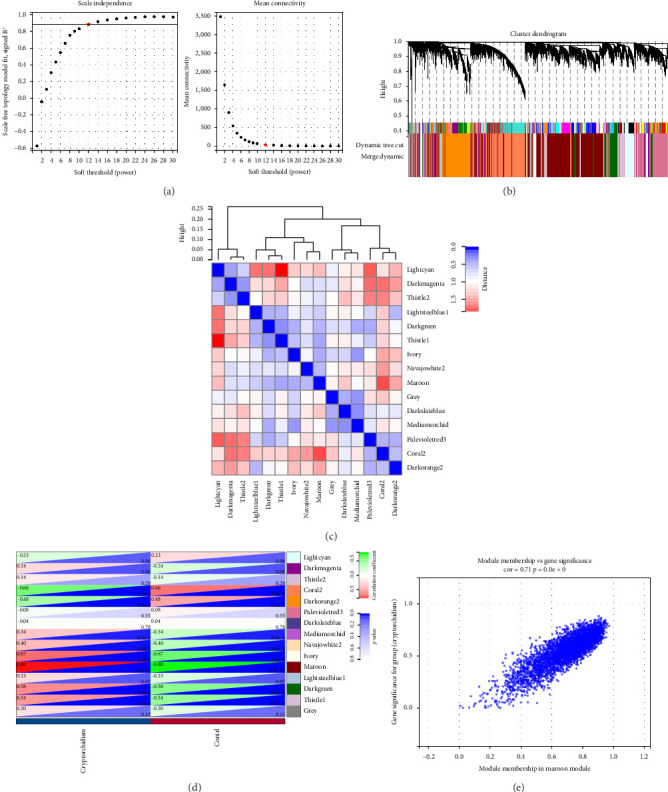
Preprocessing of WGCNA. (a) Analysis of the scale-free fit index for various soft-thresholding powers, and the mean connectivity for various soft-thresholding powers. (b) The cluster dendrogram of all genes. Each branch represents a gene and each color represents a co-expression module. (c) Interaction relationship analysis of co-expressed genes. (d) Heatmap plot of the adjacencies in the hub gene network. (e) Gene significance (GS) for weight versus module membership (MM) in maroon module.

WGCNA co-expression network was constructed to identify modules strongly associated with cryptorchidism. We chose 12 as the power and 0.89 as the scale-independent value. Under these circumstances, the average connectivity was high, and the connectivity between genes was in accordance with the scale-free network distribution (Figure 3(a)). Then, on the basis of mean linkage clustering, all of the genes with analogous expression patterns were divided into different modules (Figure 3(b)). To further confirm the correlations among these modules, the heatmap were analyzed (Figure 3(c)). Finally, 15 modules were identified with a distance of less than 0.25. Notably, the maroon module (*r* = 0.85 *p* = 0.0e + 0), containing 2369 genes, was obviously associated with disease conditions, indicating that these genes in the maroon module may play a critical role in the pathogenesis of cryptorchidism (Figure 3(d)). In addition, there was a highly significant correlation between gene significance (GS) for cryptorchidism and module membership (MM) in the maroon module (Figure 3(e)).

### 3.3. Identification of DE-FRGs

**Figure 4:**
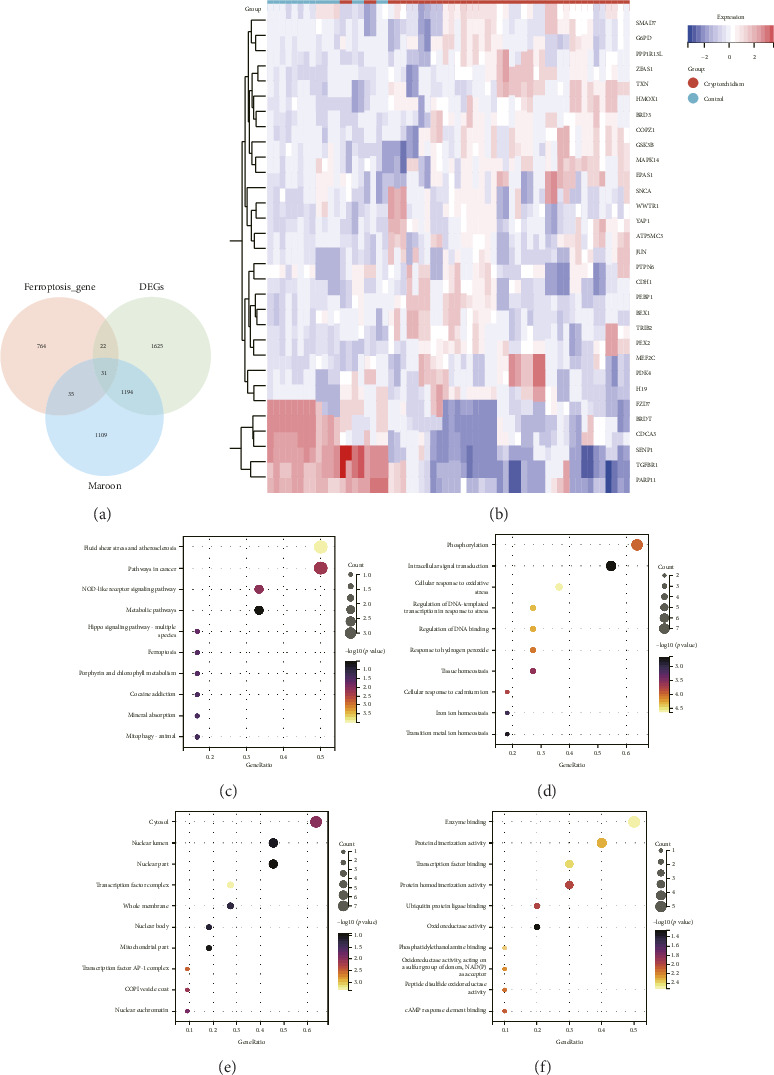
Identification of DE-FRGs and GO and KEGG analysis. (a) A Venn diagram was used to obtain the intersection genes of the three methods. (b) Heatmap for 31 DE-FRGs. (c) The results of the KEGG analysis. (d, e, f) The results of the GO analysis, including biological processes, cell components, and molecular functions. The size of the dot represents the number of gene counts, and the color of the dot represents the *p* value.

The data of 852 genes from the FerrDb v2 database were crossed with the DEGs from the meta datasets of GSE16191, GSE25518 and E_TABM_1214 and the genes of the maroon module to determine DE-FRGs. In total, 26 upregulated genes and 5 downregulated genes were documented (Figure 4(a)). The heatmap of these 31 DE-FRGs are shown in Figure 4(b) and details are listed in Table 1. These DE-FRGs were related to ferroptosis driver, suppressor, or marker genes.

### 3.4. GO and KEGG Enrichment Analysis of DE-FRGs

To study the potential biological functions and signaling pathways of the DE-FRGs, we performed GO and KEGG enrichment analyses. In the KEGG pathway enrichment analysis, the DE-FRGs were associated mainly with hippo signaling pathway-multiple species, ferroptosis and mitophagy-animal (Figure 4(c)). The GO analysis results revealed that the DE-FRGs were enriched primarily in cellular response to oxidative stress, response to hydrogen peroxide, tissue homeostasis, cellular response to cadmium ion, iron ion homeostasis and transition metal ion homeostasis (BP). Secondly, these genes are involved in the cytosol, nuclear lumen, nuclear part, transcription factor complex, whole membrane, nuclear body, mitochondrial part, transcription factor AP-1 complex and COPI vesicle coat and nuclear euchromatin (CC). Finally, the DE-FRGs were also enriched in ubiquitin protein ligase binding, oxidoreductase activity, acting on a sulfur group of donors, NAD(P)as acceptor and peptide disulfide oxidoreductase activity (MF) (Figures 4(d), 4(e), 4(f)).

### 3.5. Gene Set Enrichment and IHC Analysis

**Figure 5:**
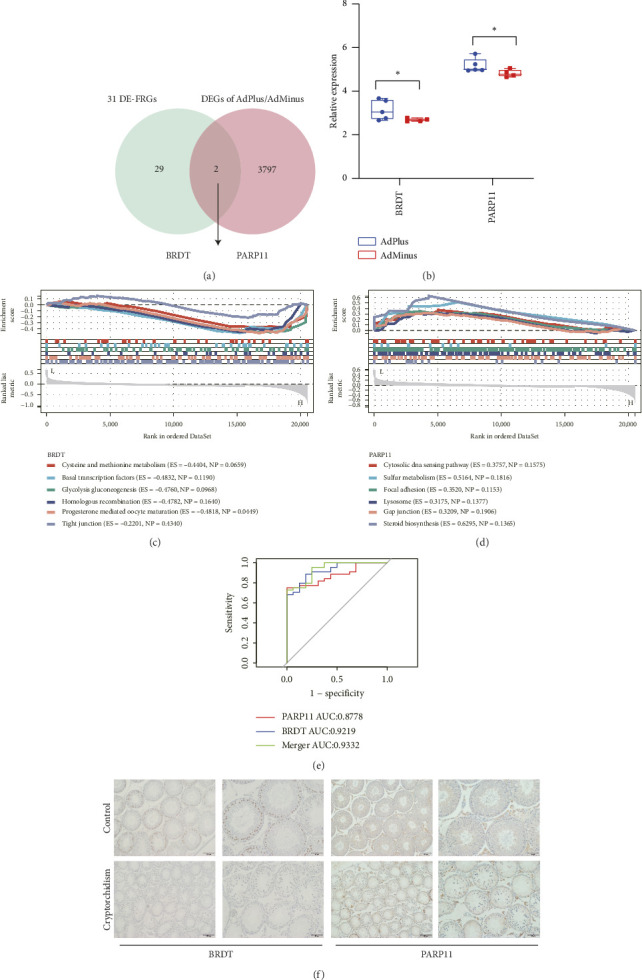
Identification of two key genes. (a) 31 DE-FRGs intersecting with DEGs in AdPlus/AdMinus. (b) Two key DEFRGs relative expression between normal controls and patients with cryptorchidism. (c, d) GSEA enrichment analysis showing signaling pathways enriched by BRDT and PARP11. (e) ROC analysis. (f) Representative images of immunohistochemical staining for BRDT and PARP11 in Rat testicular tissue.

There is a general consensus that male infertility is a major complication of cryptorchidism. To further explore the relationship between cryptorchidism and spermatogenesis, we analyzed the DEGs in AdPlus/AdMinus from the ArrayExpress database (|FC| ≥ 1.2 and *p* values < 0.05). Finally, two key genes most closely associated with spermatogenesis were identified via taking the intersection of 31 DE-FRGsand DEGs in AdPlus/AdMinus (Figure 5(a)). Both of the two genes were down-regulated in AdMinus cryptorchidism testes (Figure 5(b)), which is consistent with our hypothesis. The GSEA results revealed that the main enriched signaling pathways for high BRDT expression were progesterone mediated oocyte maturation and tight junction (TJ) (Figure 5(c)). The main enriched pathways for high PARP11 expression were lysosome, gap junction and steroid biosynthesis (Figure 5(d)). The ROC analysis revealed that the AUC values of BRDT and PARP11 were 0.9219 and 0.8778 respectively. However, when we combined these two genes for analysis, the AUC value improved to 0.9332 (Figure 5(e)). Animal experiments reveal seminiferous tubule atrophy and a reduction in testicular volume in cryptorchid testes (Supporting Figure 1). Meanwhile, BRDT and PARP11 were subsequently validated by IHC and both of the two genes expression decreased in cryptorchid tissue, which was consistent with our previous conclusions (Figure 5(f)).

### 3.6. PPI Construction of Hub Genes

**Figure 6:**
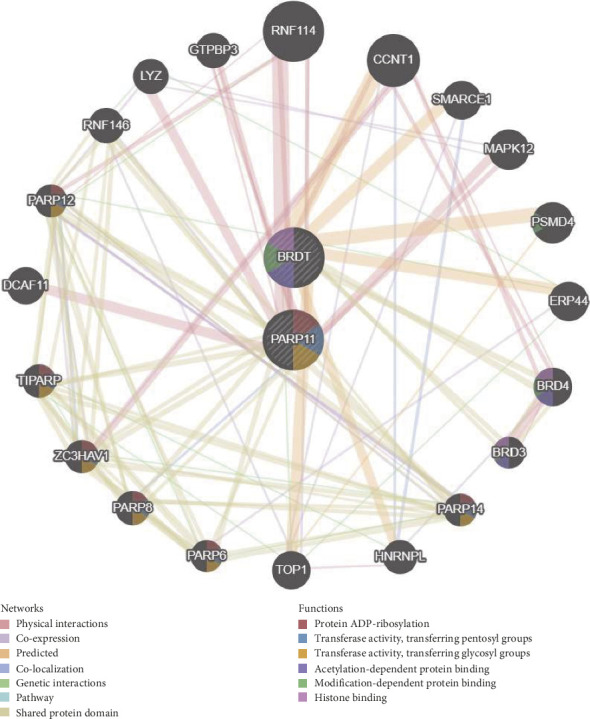
The gene–gene interaction network for DEGs was analyzed using the GeneMANIA database. The 20 most frequently changed neighboring genes are shown. The predicted genes are located in the outer circle, and hub genes are in the inner circle.

To predict interactions among physical interactions, co-expression, prediction, co-localization, genetic interactions, pathway and shared protein domain, PPI analysis of the two hub genes and the genes they interact with were performed via GeneMANIA (Figure 6). These genes were enriched mainly in protein ADP-ribosylation, acetylation-dependent protein binding, modification-dependent protein binding and histone binding.

### 3.7. Further miRNA Interaction and Enrichment

**Figure 7:**
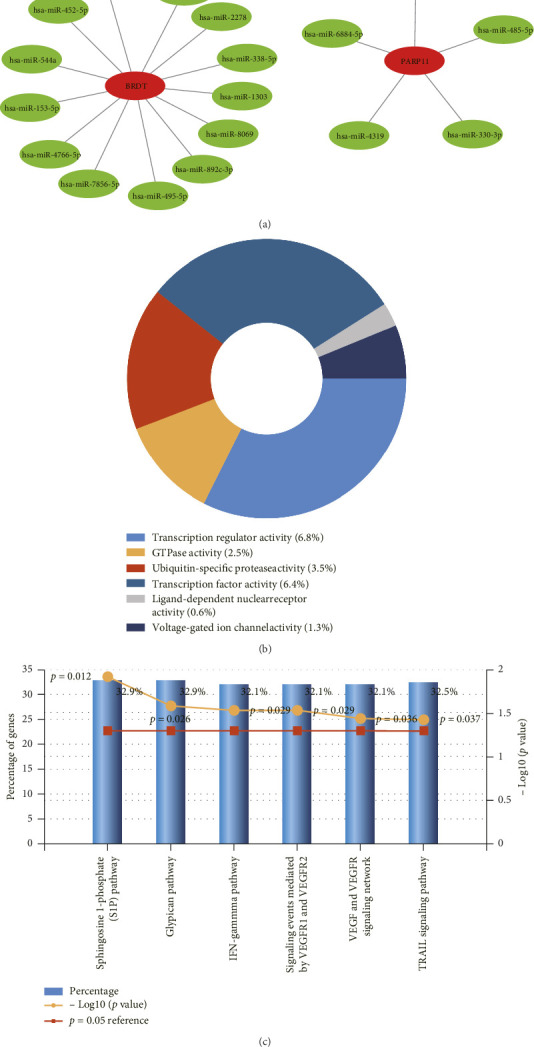
Interaction networks between key DEFRGs and their targeted miRNAs. (a) Interaction network between genes of the key DEFRGs and its targeted miRNAs. Genes were colored in red, and miRNAs were colored in green. (b) The molecular function of key DEFRGs-related miRNAs. (c) The biological pathways enriched of the key DEFRGs-related miRNAs.

In our study, gene–miRNA analysis of the 2 key DE-FRGs associated with cryptorchidism was further examined via the miRWalk2.0 software. The correlative miRNAs were identified via the miRWalk and TargetScan databases. Finally, we selected 18 miRNA expression genes (Figure 7(a)). FunRich software was used for enrichment analysis of the key DE-FRGs-related miRNAs. The molecular function significantly enriched in the transcription regulator activity (6.8%), GTPase activity (2.5%), ubiquitin-specific protease activity (3.5%), transcription factor activity (6.4%), ligand-dependent nuclear receptor activity (0.6%), voltage-gated ion channel activity (1.3%; Figure 7(b)). The sphingosine 1-phosphate (S1P) pathway (32.9%), glypican pathway (32.9%), and TRAIL signaling pathway (32.5%) were the top 3 biological pathways identified (Figure 7(c)).

## 4. Discussion

Cryptorchidism is one of the most common congenital anomalies encountered in pediatric patients and a pathological condition in which the testis fails to descend to the base of the scrotum. Hormonal, genetic and environmental factors might also be etiological factors of cryptorchidism. The influence of cryptorchidism on male infertility is well known.

Ferroptosis is mainly caused by intracellular iron overload and lipid ROS accumulation, which plays a significant role in physiological and pathogenic processes [27]. Ferroptosis can reportedly lead to male reproductive dysfunction [28]. The molecular mechanisms of ferroptosis inducing spermatogenesis dysfunction need to be further explored, but several pathways and genes related to ferroptosis are closely related to spermatogenesis dysfunction [29]. For example, the spermatocyte-specific GPX4 knockout male mice presented a decrease in the number of spermatozoa and infertility [30]. However, the relationship between the molecular mechanisms of spermatogenesis dysfunction in cases of cryptorchidism and ferroptosis has never been reported. Therefore, exploring the pathogenesis of spermatogenesis dysfunction in patients with cryptorchidism caused by ferroptosis may provide new perspectives on the prevention and treatment of infertility in patients with cryptorchidism.

In this study, we demonstrated an association between spermatogenesis dysfunction and ferroptosis in patients with cryptorchidism. We identified 31 DEFRGs on the basis of the GEO and ArrayExpress databases and found that they were enriched mainly in the hippo signaling pathway-multiple species, ferroptosis and mitophagy-animal in KEGG pathway. These DE-FRGs are closely linked with ferroptosis, and hippo signaling pathway and mitophagy are also closely linked to embryogenesis development [31]. Similarly, the results of the GO analysis indicated these genes were mainly enriched in cellular response to oxidative stress, response to hydrogen peroxide, tissue homeostasis, cellular response to cadmium ion, iron ion homeostasis and transition metal ion homeostasis (BP). These genes were also enriched in the cytosol, nuclear lumen, nuclear part, transcription factor complex, whole membrane, nuclear body, mitochondrial part, transcription factor AP-1 complex and COPI vesicle coat and nuclear euchromatin (CC). Moreover, these genes were also involved in ubiquitin protein ligase binding, oxidoreductase activity, acting on a sulfur group of donors, NAD(P)as acceptor and peptide disulfide oxidoreductase activity (MF). The above results suggest that these genes may be involved in a range of biological process, such as ferroptosis and oxidative stress and may play a key role in spermatogenesis in patients with cryptorchidism. In addition, we confirmed two key DE-FRGs, BRDT and PARP11, by integrating the DEGs associated with spermatogenesis, which were downregulated in AdMinus testes of patients with cryptorchidism. The GSEA results revealed that the main enriched signaling pathways for high BRDT expression were progesterone mediated oocyte maturation and TJ and the main enriched pathways for high PARP11 expression were lysosome, gap junction and steroid biosynthesis. These results suggest that these two genes may play an essential role in lysosomes and connections between cells. The BTB is generated by multiple junctions between Sertoli cells, including TJs, adherens junctions, and other junctional complexes, which maintain a unique microenvironment for normal spermatogenesis [32]. We speculated that these genes may take part in maintaining BTB integrity and future contribute to spermatogenesis. Furthermore, we conducted PPI analysis of the two hub genes and their 20 interacting genes to predict correlations among co-localization, shared protein domains, co-expression, prediction and pathways and found the functions of these genes were enriched mainly in protein ADP-ribosylation, acetylation-dependent protein binding, modification-dependent protein binding and histone binding. Many studies have indicated that these functions are directly or indirectly associated with spermatogenesis [33–35]. Finally, we predicted the miRNAs interacted with these two genes, and found they weremainly enriched in sphingosine 1-phosphate (S1P) pathway, glypican pathway and TRAIL signaling pathway. Suomalainen et al. suggested that S1P inhibits male germ cell apoptosis independently of its receptors, possibly by inhibiting the transcription factor NF-kappaB and Akt phosphorylation [36]. Moreover, a study indicated that TRAIL is an important signaling molecule for maintaining germ cell homeostasis and functional spermatogenesis in the testis [37].

Hadziselimovic et al. reported that cryptorchid cases in young men had presented expression of most of the genes essential for spermatogenesis via whole genome expression profiling of testicular tissue [38]. Moreover, testicular descent is regulated by hormones. Many investigations suggest that normal testicular descent requires a normally functioning hypothalamic-pituitary-testicular axis [39]. Thermal insult damage increases the risk of both cancer and infertility in congenital undescended testes [40]. Research on the effects of heat stress on mammalian male germ cells has focused on the following aspects: germ cell apoptosis and sperm DNA damage. Moreover, Sertoli and Leydig cells respond to heat stress, and the expression of some genes in male germ cells is also affected by heat stress. For example, a few genes that are possibly related to apoptotic processes are upregulated, whereas genes for DNA repair and cell cycle regulation are downregulated after heat stress [41].

Research has shown that the bromodomain testis-specific (BRDT) protein is a part of the bromodomain extra-terminal (BET) family of proteins [42]. Moreover, it is expressed specifically in the testis [2, 12, 33, 43–45], and serves as a transcriptional regulator of gene expression during spermatogenesis [46]. Barda S. et al. confirmed that mice lacking the BRDT gene were sterile with a complete absence of spermatozoa in the epididymis, and their continuous mating with fertile females failed to achieve any pregnancy [47].

Enzymes of the PARP family cleave nicotinamide adenine dinucleotide (NAD) into nicotinamide and ADP-ribose,then, transfering the ADP-ribose unit onto specific amino acid side chains of target proteins as a posttranslational modification [48]. Both PARP1, the archetypal enzyme of the family, and PARP2 are well known to be involved in the regulation of DNA repair, chromatin dynamics, and many other cellular processes [49–53]. Meyer-Ficca et al. demonstrated that a lack of PARP11 in knockout animals caused teratozoospermia with nuclear membrane abnormalities and resulted in male infertility [54].

These findings suggest that BRDT and PARP11 are associated with apoptosis and spermatogenesis, which is consistent with our findings. Overall, we deduced that BRDT and PARP11 downregulation in patients with cryptorchidism may hinder spermatogenesis, which could be used as a reference for early therapeutic strategies for patients with cryptorchidism. In addition, cryptorchidism patients are mostly treated with testicular descent and fixation in clinical practice, and our findings may provide a useful reference for predicting whether spermatogenesis dysfunction occurs in cryptorchidism patients after surgery. Furthermore, our findings suggest that ferroptosis may play an important role in spermatogenesis dysfunction due to cryptorchidism, which has rarely been previously reported. Hence, our findings provide a new research direction for investigating spermatogenesis dysfunction in patients with cryptorchidism in clinical practice.

In the meantime, this study has several limitations. First, our research results were based solely on bioinformatics analysis, and further basic and clinical experiments are needed to verify these results. Second, the sample size included in our study was not large enough due to the difficulty in collecting cryptorchid samples, which may have led some deviations in the final results. Furthermore, more ferroptosis-related genes were discovered as the FerrDb database was updated. Therefore, the research results may deviate from the actual situation.

## 5. Conclusion

In conclusion, our study revealed that BRDT and PARP11 were downregulated in AdMinus testes of cryptorchidism, which suggested that ferroptosis might play a significant role in spermatogenic dysfunction in cryptorchidism. Meanwhile, this study provided new perspectives on the prevention and treatment of the infertility in patients with cryptorchidism and potential future research directions for investigating spermatogenesis dysfunction in patients with cryptorchidism.

## Figures and Tables

**Table 1:** 31 DE-FRGs of cryptorchidism.

Symbol	FC	*p* value
ATP5MC3	1.540935482	4.16496E − 12
G6PD	2.316009269	1.06866E − 07
HMOX1	1.938507556	1.92837E − 12
PEBP1	1.61266207	1.03608E − 08
MAPK14	1.575362044	1.15387E − 05
EPAS1	1.51165769	1.01433E − 08
PEX2	1.626725712	2.81075E − 12
SMAD7	1.794757347	1.48441E − 05
GSK3B	1.53960154	1.01317E − 06
ZFAS1	1.861678078	1.3135E − 11
SNCA	1.620948543	2.92463E − 08
WWTR1	1.634941954	4.92593E − 08
H19	2.39470499	2.46221E − 08
YAP1	1.741969592	2.13713E − 07
PTPN6	1.826618974	1.56255E − 08
JUN	1.889369838	5.70819E − 10
FZD7	1.799230546	8.66196E − 09
CDH1	2.307137809	3.95817E − 07
BRD3	1.554473758	4.03504E − 10
PPP1R13L	2.153406268	6.32167E − 09
COPZ1	1.622352457	7.84107E − 16
TXN	1.62241432	1.85018E − 10
BEX1	1.87065214	2.76695E − 08
MEF2C	2.050909383	4.49519E − 11
TRIB2	1.90593396	1.58421E − 11
PDK4	1.642245867	1.3736E − 07
TGFBR1	0.439499795	1.24434E − 07
CDCA3	0.592679908	4.24288E − 06
BRDT	0.259147716	1.95662E − 08
PARP11	0.635630828	1.894E − 07
SENP1	0.582180541	1.4773E − 05

## Data Availability

The data that support the findings of this study are available from the corresponding authors upon reasonable request.

## Supporting Information

Supporting Figure 1: Pathological changes of cryptorchidism. (A) The overall morphology of the testes in normal and cryptorchid rats. (B) H&E staining results of testes from normal and cryptorchid rats.
