# Supplementary figures and images for: Identification of Ferroptosis-Related Genes Associated With Cryptorchidism via Bioinformatics and Experimental Verification
Source: Genet Res (Camb). 2025 May 23;2025:7355474. doi: 10.1155/genr/7355474 (PMC12124929; doi:10.1155/genr/7355474)

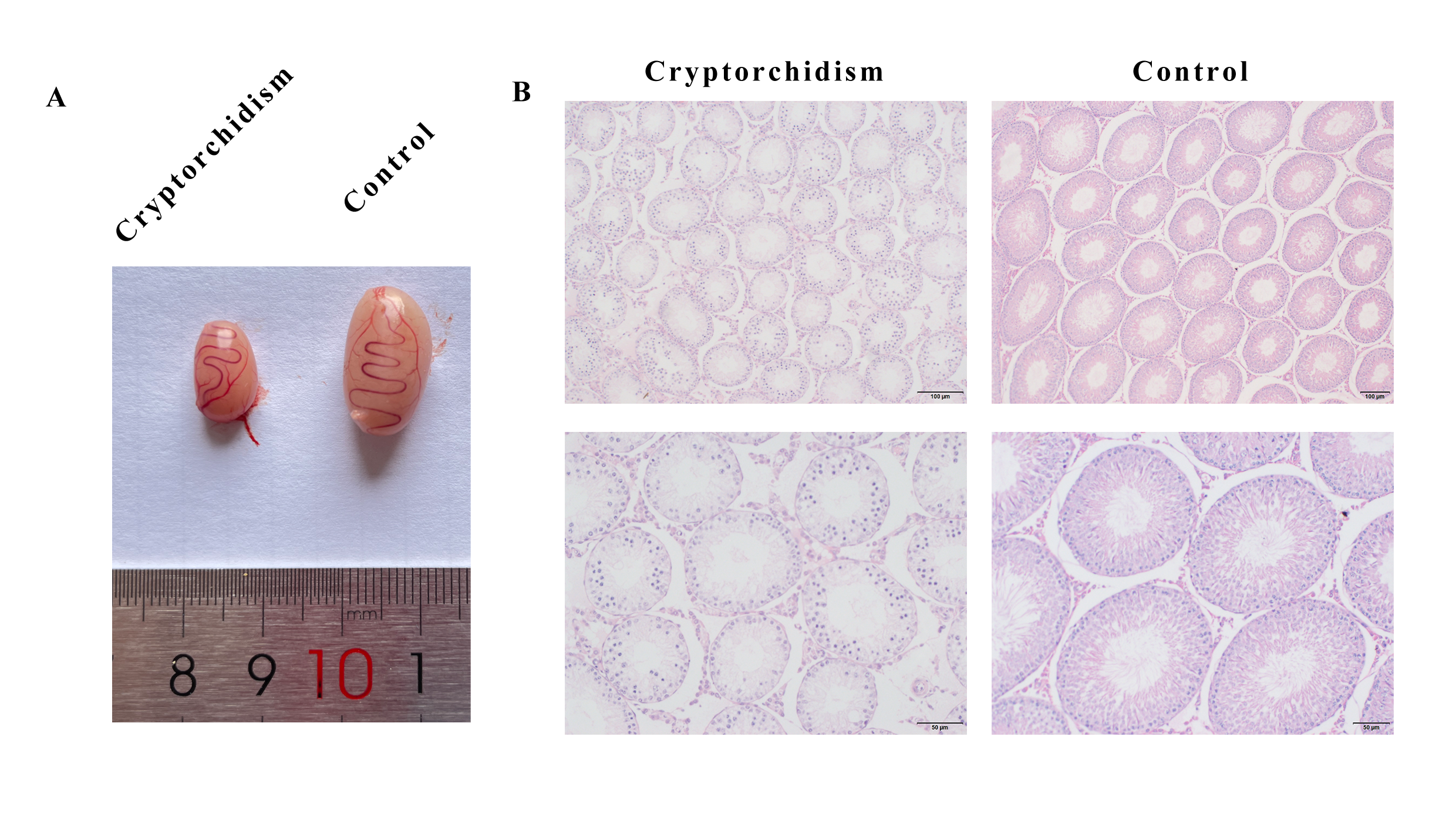

Supplement: Supporting Information — Additional supporting information can be found online in the Supporting Information section. [file 7355474.f1.tif]
